# Supplementary material for: PUFFFIN: an ultra-bright, customisable, single-plasmid system for labelling cell neighbourhoods
Source: EMBO J. 2024 Jul 12;43(18):14. doi: 10.1038/s44318-024-00154-w (PMC11405414; doi:10.1038/s44318-024-00154-w)
Supplement: Supplementary file 4 — Expanded View Figures [file 44318_2024_154_MOESM4_ESM.pdf]

Expanded View Figures

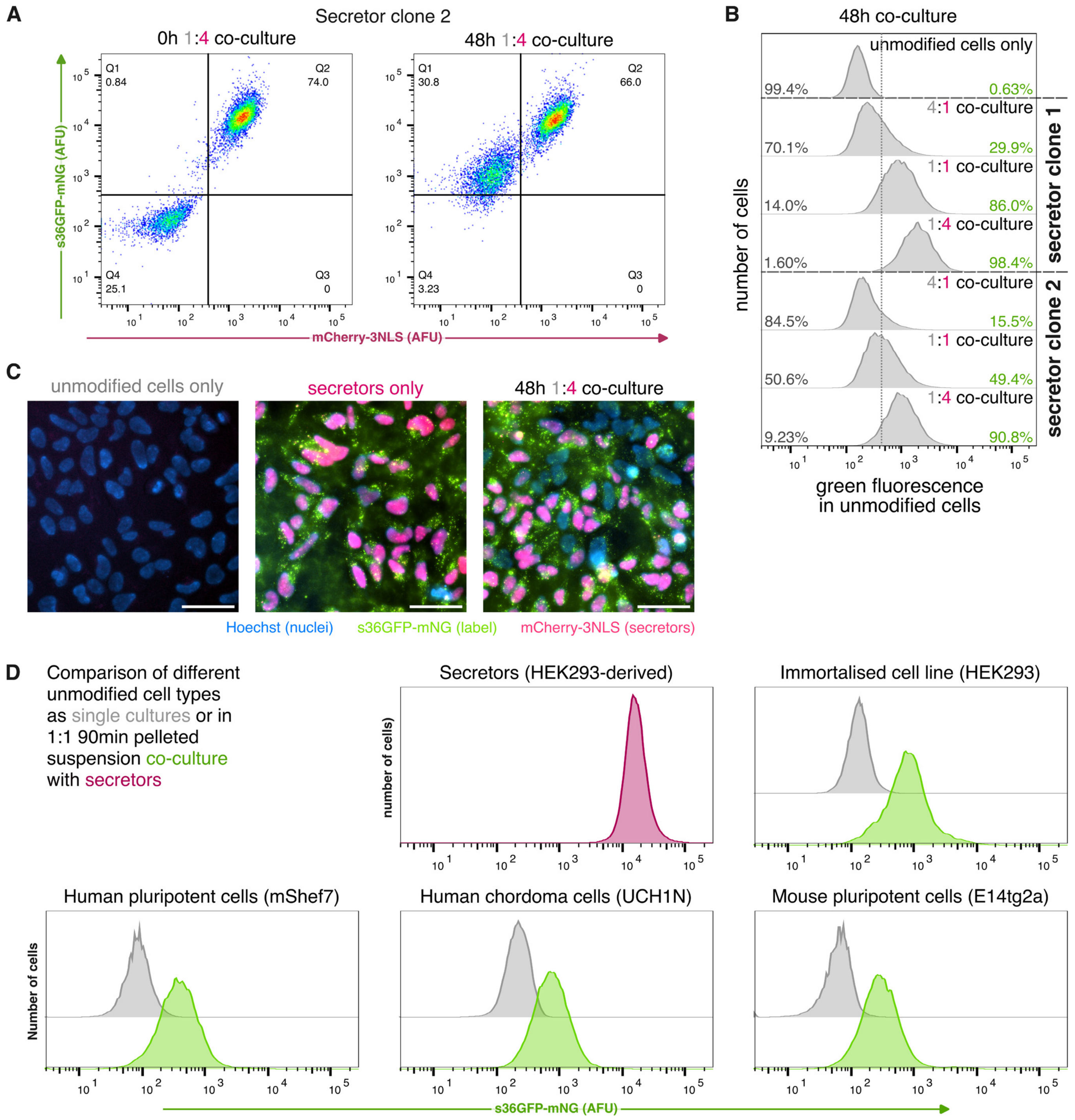

Figure EV1. Secretors can effectively label unmodified cells by transferring s36GFP-mNG.

(**A**) Flow cytometry of a 1:4 co-culture of unmodified cells and a second independent monoclonal secretor line (secretor clone 2) at 0 and 48 h time points. Three independent experiments were performed, and a representative set is shown. (**B**) Flow cytometry of a 48 h co-culture with unmodified cells and either secretor clone 1 or secretor clone 2 seeded at different co-culture ratios. Grey numbers (bottom left of each plot) are the percentage of unmodified cells in Q4, and green numbers (bottom right of each plot) are the percentage of unmodified cells in Q1. Three independent experiments were performed, and a representative set is shown. (**C**) Live imaging of single cultures of unmodified cells or secretors and a 48 h 1:4 co-culture of unmodified cells and secretors with Hoechst nuclear staining. The scale bar is 50 µm. (**D**) Flow cytometry of 1:1 90 min pelleted suspension co-cultures of different unmodified cell types and HEK293-derived secretors.

 © The Author(s)

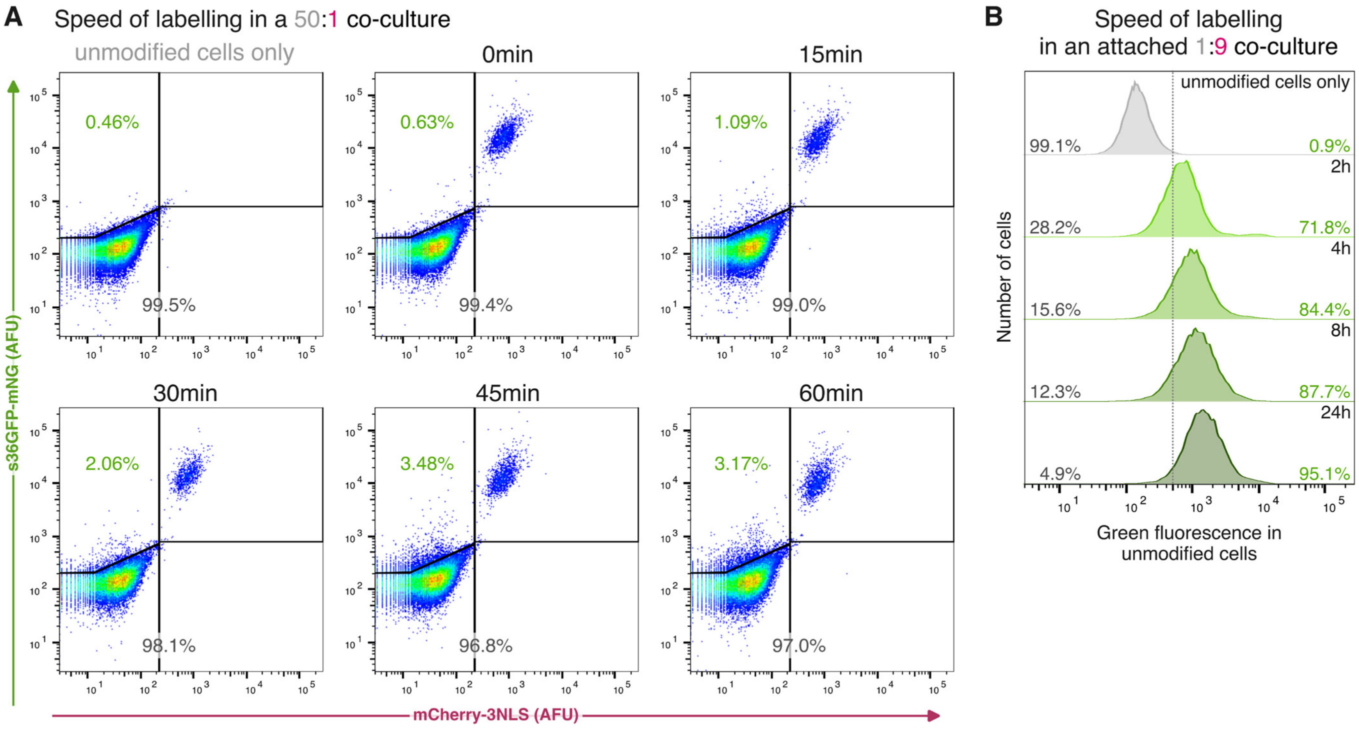

Figure EV2. PUFFFIN labelling is time-dependent and fast.

(**A**) Speed of labelling with few secretors by flow cytometry analysis of different 50:1 unmodified cell:secretor ratios in a pelleted suspension co-culture as a time course experiment. Grey numbers are percentage of unmodified cells in top polygon, green numbers are percentage of unmodified cells in bottom polygon. 50,000 cells were analysed for each sample. Three independent experiments were performed, and a representative set is shown. (**B**) Speed of labelling in an attached monolayer co-culture shown by flow cytometry analysis of a 1:9 unmodified cell:secretor co-culture time course experiment. 10,000 unmodified cells were analysed for each sample. Grey numbers (bottom left of each plot) are the percentage of unmodified cells in Q4, and green numbers (bottom right of each plot) are the percentage of unmodified cells in Q1. Three independent experiments were performed, and a representative set is shown.

© The Author(s)

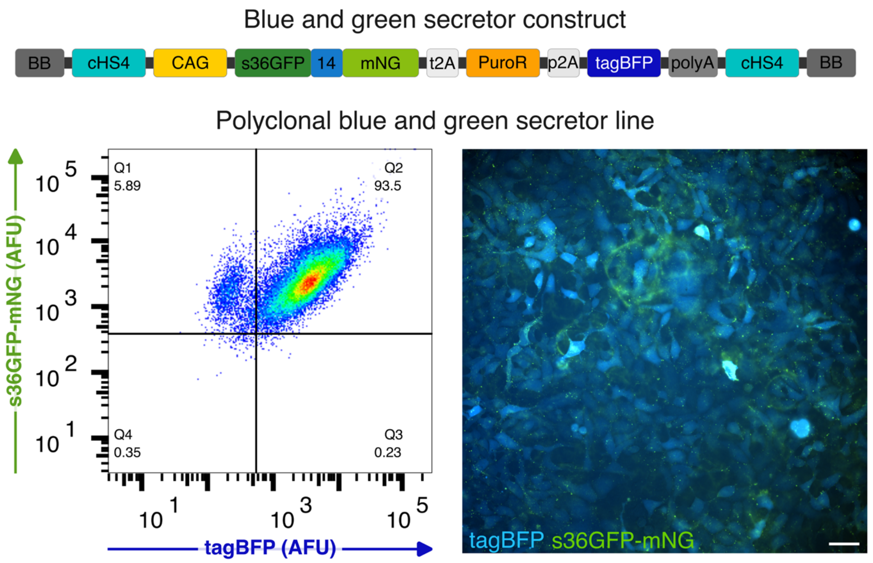

Figure EV3. Modularity of the PUFFFIN expression construct allows change of colour for the secretor-cell-marker.

Polyclonal secretors expressing s36GFP-mNG and cytoplasmic tagBFP made by random integration of the blue-green secretor construct (top) are shown by flow cytometry (left) and imaging live cells for green and blue fluorescence (right). The scale bar is 50 µm.

 © The Author(s)

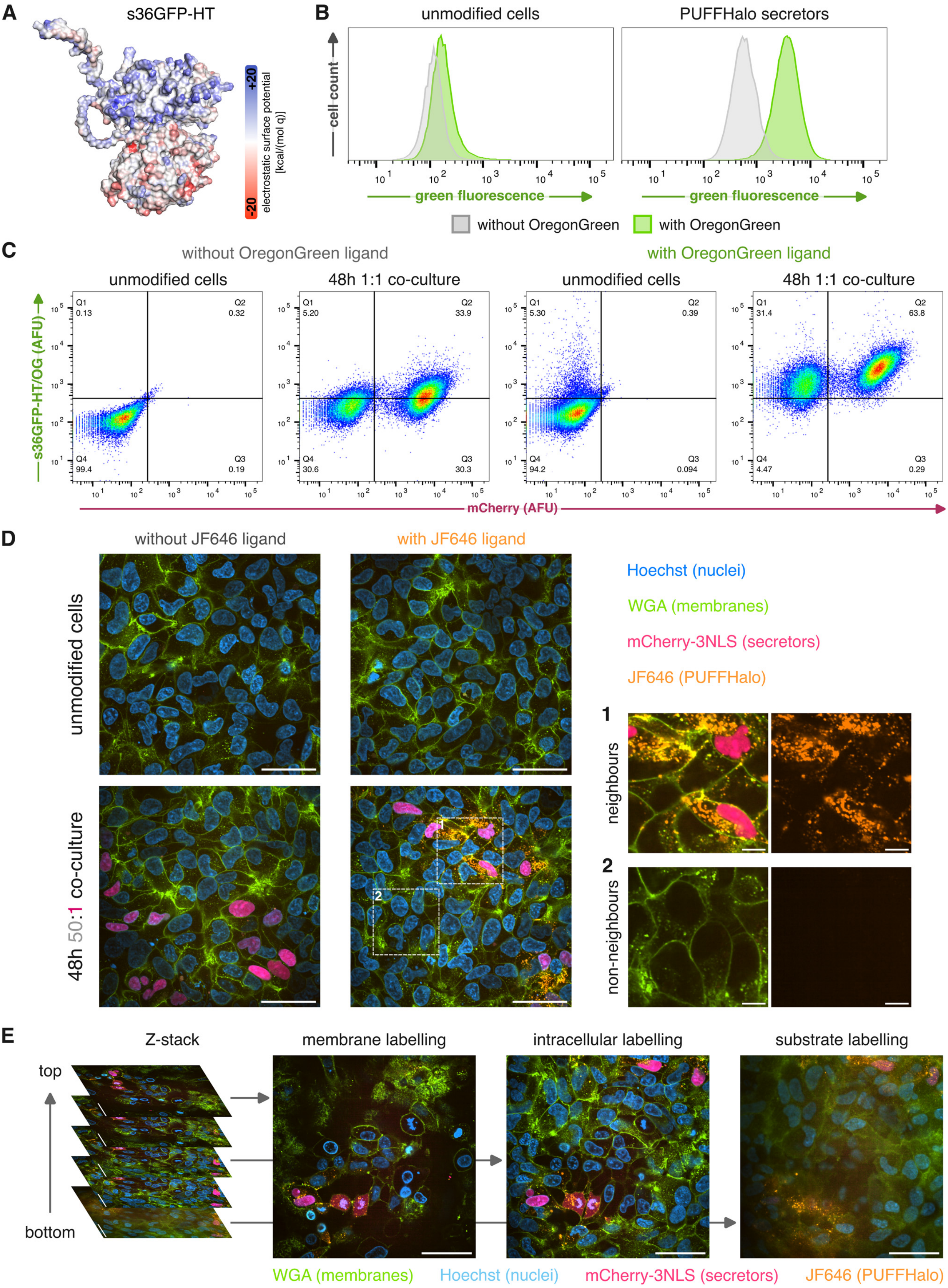
A
s36GFP-HT
+20
-20
electrostatic surface potential [kcal/(mol q)]
B
unmodified cells
PUFFHalo secretors
cell count
green fluorescence
without OregonGreen
with OregonGreen
C
without OregonGreen ligand
with OregonGreen ligand
unmodified cells
48h 1:1 co-culture
s36GFP-HT/OG (AFU)
mCherry (AFU)
D
without JF646 ligand
with JF646 ligand
unmodified cells
48h 50:1 co-culture
Hoechst (nuclei)
WGA (membranes)
mCherry-3NLS (secretors)
JF646 (PUFFHalo)
neighbours
non-neighbours
E
Z-stack
membrane labelling
intracellular labelling
substrate labelling
top
bottom
WGA (membranes)
Hoechst (nuclei)
mCherry-3NLS (secretors)
JF646 (PUFFHalo)

Figure EV4. PUFFHalo integrates HaloTag technology with PUFFFIN for choice-of-colour labelling.

(**A**) Electrostatic surface potential is shown for s36GFP-HaloTag (AlphaFold predicted structure). (**B**) Unmodified cells and PUFFHalo secretors were incubated for 2 h with or without the HaloTag ligand OregonGreen. Three independent experiments were performed, and a representative set is shown. (**C**) Flow cytometry of 48 h 1:1 co-cultures of unmodified cells and PUFFHalo secretors were incubated for 2 h with or without the HaloTag ligand OregonGreen. Three independent experiments were performed, and a representative set is shown. (**D**) Live imaging of single cultures of unmodified cells and 48 h 50:1 co-cultures of unmodified cells and PUFFHalo secretors with or without the HaloTag ligand JF646, all with Hoechst nuclear staining and WGA488 membrane staining. Scale bars are 50 µm. Two regions of the 48 h 1:1 co-culture with JF646 are magnified to show neighbours and non-neighbours. Scale bars of (1) and (2) are 10 µm. (**E**) Three single planes of a Z-stack for a 48 h 1:1 co-culture with JF646, WGA488, and Hoechst staining were selected to highlight different localisations of the s36GFP-HT label. Scale bars are 50 µm.

© The Author(s)

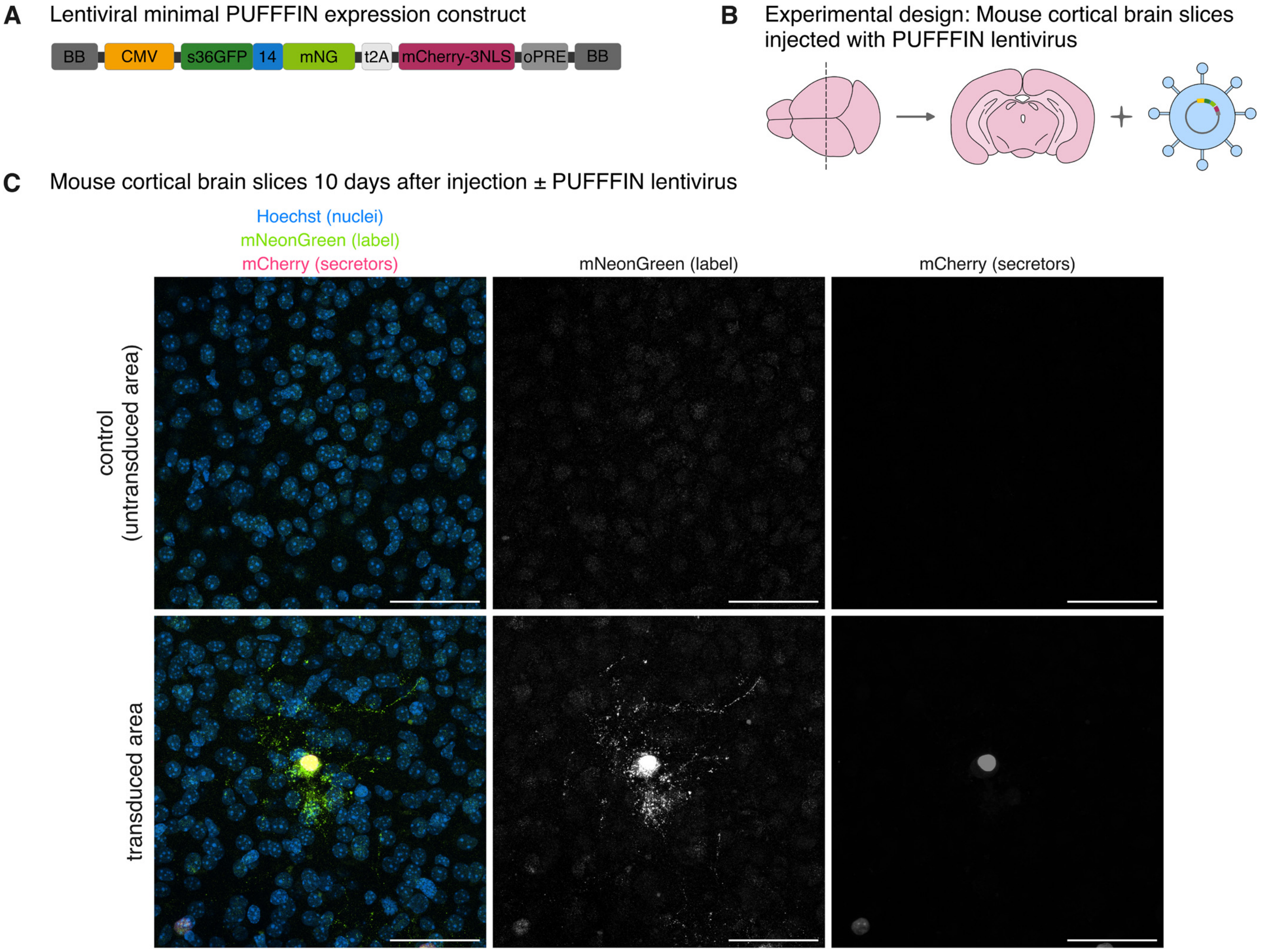

Figure EV5. PUFFFIN expression in mouse organotypic brain slices using a lentiviral vector.

(**A**) Minimal PUFFFIN expression construct as used in lentivirus vector. (**B**) Illustration of the experimental design for transducing cultured mouse cortical slices with the PUFFFIN lentivirus. (**C**) Immunofluorescence of mouse cortical organotypic slices untransduced or transduced with 8.9×10^6 TU/ml of PUFFFIN lentivirus, cultured for 10 days after injection, and stained for mNeonGreen and mCherry, and counterstained with Hoechst. Scale bars are 50 µm.

© The Author(s)

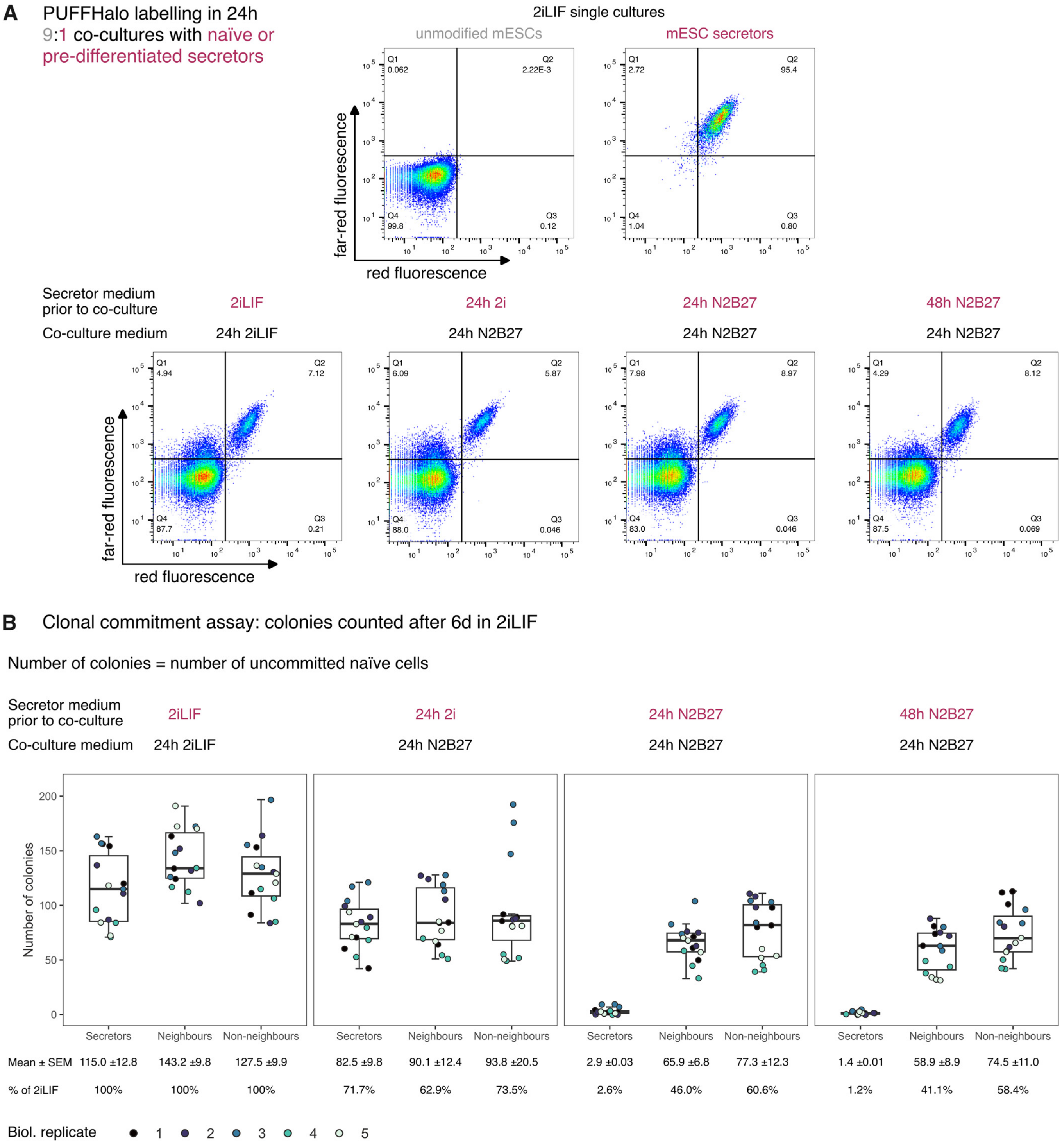

Figure EV6. PUFFHalo labelling in mESCs can be utilised for investigating exit from pluripotency.

(**A**) PUFFHalo labelling shown for 2iLIF single cultures and 24 h 9:1 co-cultures in different media conditions shown by flow cytometry. Three independent experiments were performed, and a representative set is shown. (**B**) Colony formation efficiency (CFE) is shown as the number of colonies for the clonal commitment assay counted after 6 days in 2iLIF, coloured by biological replicate. Boxes denote data within the 25th and 75th percentiles, the middle line represents the median, and the whiskers extend from the upper and lower quartiles to a distance of 1.5 times from the median. Below the plot, mean colony numbers ± standard error of the mean (SEM) and percentages relative to 2iLIF are reported for secretors, neighbours and non-neighbours. Five independent experiments with three non-independent replicates were performed.

 © The Author(s)
